# Invasive Lobular Carcinoma in Premenopausal Woman: A Delayed Diagnosis Due to Socio-Cultural Factors Prevalent in Pakistan

**DOI:** 10.7759/cureus.24766

**Published:** 2022-05-05

**Authors:** Shafi Rehman, Jai Sivanandan Nagarajan, Bushra Ghafoor, Muhammad Hamza Qureshi, Shazmah Shahrukh

**Affiliations:** 1 Internal Medicine, Lady Reading Hospital, Peshawar, PAK; 2 Internal Medicine, SRM Medical College Hospital and Research Centre, Chennai, IND; 3 Internal Medicine, Nishtar Medical University, Multan, PAK; 4 Internal Medicine, Mayo Hospital, Lahore, PAK; 5 Internal Medicine, Dow University of Health Sciences, Civil Hospital Karachi, Karachi, PAK

**Keywords:** reluctance to see male physician, premenopausal woman, cost of breast cancer treatment, mastectomy, financial, stigmatization, religious misconception, health education & awareness, socio- cultural factors, invasive lobular breast carcinoma

## Abstract

A 41-year-old premenopausal woman presented to the outpatient department with a diagnosis of invasive lobular carcinoma. She noticed a lump a year back but did not seek medical attention due to many socio-cultural factors prevalent in Pakistan that prevent her from seeking medical attention earlier. She came in for a check-up after increasing in size of the lump. The bilateral mammogram showed large areas of asymmetrical density in the left upper quadrant. It was followed by an ultrasound-guided biopsy which confirmed the diagnosis of invasive lobular carcinoma. Due to stage 3, it was recommended to have CT and an MRI of the breast to assess the extent of the disease. Tissue immunohistochemistry was also requested, which came back as ER-positive, PR-positive, and HER2/neu negative. MRI of the breast revealed a 4.2 x 3.3cm heterogeneously enhancing asymmetric mass-like enhancement area within the left breast outer quadrant with an adjacent spiculated nodular lesion measuring 2.2 cm. CT chest/abdomen/pelvis with contrast showed left breast with minimal parenchymal asymmetry and a small 9 mm node seen in the left axilla. There was no evidence of metastasis. The patient was started on neoadjuvant therapy to minimize systemic disease, followed by mastectomy. This case highlights socio-cultural factors prevalent in Pakistan that lead to delays in the diagnosis and treatment of invasive lobular carcinoma. The outcome had been better if the patient sought medical attention sooner at an earlier stage. We also propose strategies to raise awareness in Pakistan for the timely diagnosis and treatment of breast cancer.

## Introduction

Invasive lobular carcinoma accounts for 15% of all invasive breast cancers and is the second most common type of breast cancer after invasive ductal carcinoma. Invasive Lobular Carcinoma usually presents with a lower histological grade and most of these cancers are estrogen (ER) positive. They exhibit a good response to endocrine therapy and this responsiveness to treatment makes a prognosis favorable [1]. If the location of invasive breast cancer is in the breast, the five-year survival rate is 99%. If cancer has spread to the adjacent lymph nodes, the five-year survival rate drops to 86%. If cancer metastasizes to distant body parts, the five-year survival rate is only 29% [2]. It means the earlier the stage in which breast cancer is diagnosed generally the better will be its prognosis. Delay in diagnosing and treating breast cancer is a dilemma in Pakistan. Many socio-cultural factors prevalent in our system prevent women from getting timely access to a healthcare facility. This leads to delays in diagnosis and treatment of breast cancer and, consequently, a rise in the incidence rate among Asian countries [3]. We present a case of delayed diagnosis of invasive lobular carcinoma in a 41-year-old premenopausal young woman who presented late to the outpatient department due to a lack of awareness, religious misconceptions, stigmatization of breast cancer, reluctance to see a male physician, and the high cost of cancer treatment in Pakistan.

## Case presentation

A 41-year-old premenopausal Pakistani woman presented to the outpatient department of Hematology/Oncology with diagnosed invasive lobular carcinoma on biopsy. The patient described a small lump appearing over her left breast a year ago which got noticed accidentally. She did not report it to her family and physician due to lack of awareness, religious misconceptions, stigmatization of breast cancer, reluctance to see a male physician, and the high cost of cancer treatment in Pakistan. It was painless and was not associated with any other symptom. The lump grew suddenly in the last two months, which led her to visit her primary physician. She was the mother of three children delivered via vaginal delivery and breastfed for two years. She was a non-smoker; her body mass index was 30 kg/m^2^, and her menstrual cycle was regular. She had no past medical history. She was not on any medications. Her family history did not report any cancers. She also reported no weight loss. Physical examination upon arrival was deferred because the patient and her family were reluctant due to their cultural norms and religious views about consulting a male physician. Her vital signs were normal. Initial laboratory findings were normal. Inflammatory markers were not elevated.

**Figure 1:**
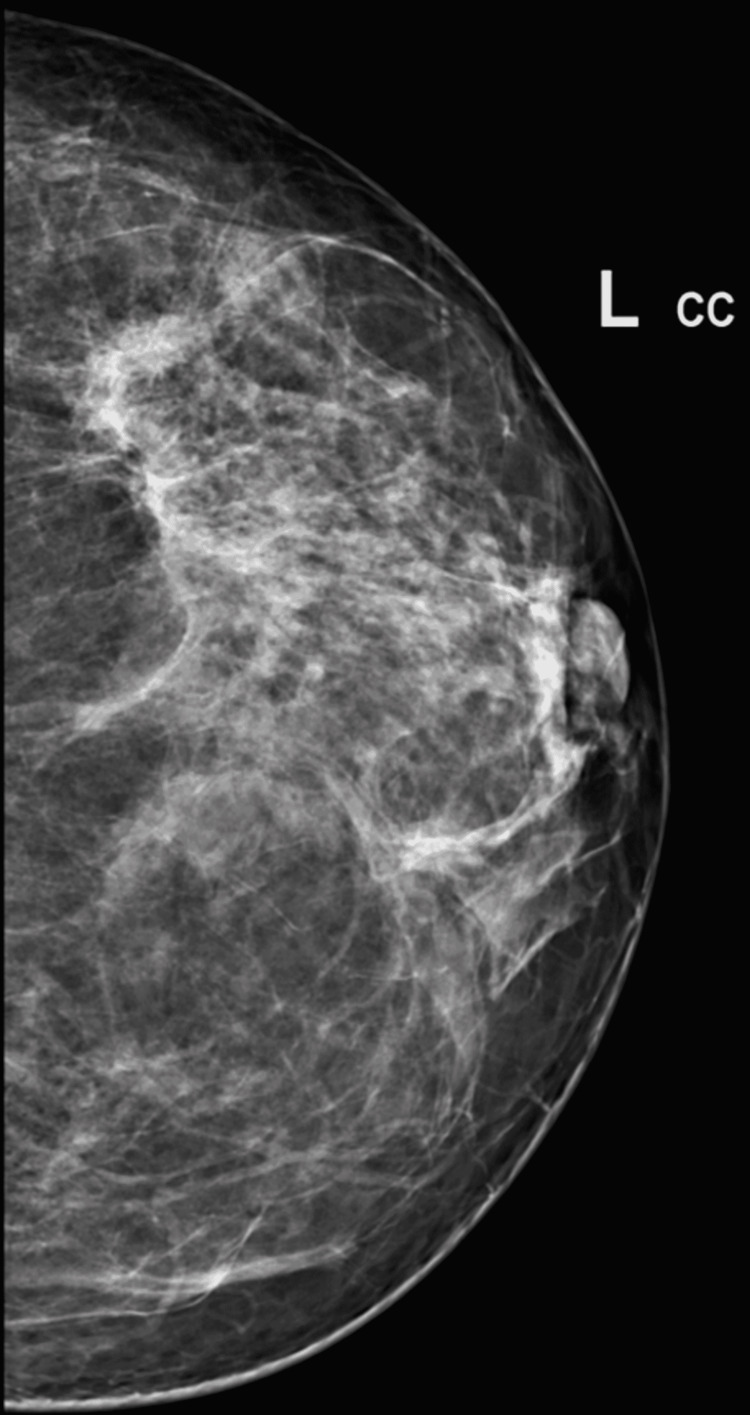
Mammography shows a large area of asymmetrical density in the left upper outer quadrant

Sono-mammography revealed that the left breast showed a large irregularly outlined hypoechoic area casting dense posterior shadowing measuring approximately 20 x 17 mm underlying the lump in the upper outer quadrant of the breast suspicious of malignant etiology and a few scattered cystic areas in the right breast parenchyma. On digital mammography, as seen in Figure 1, the left breast showed a large area of asymmetrical density in the left upper outer quadrant, which was highly suspicious of malignancy.

**Figure 2:**
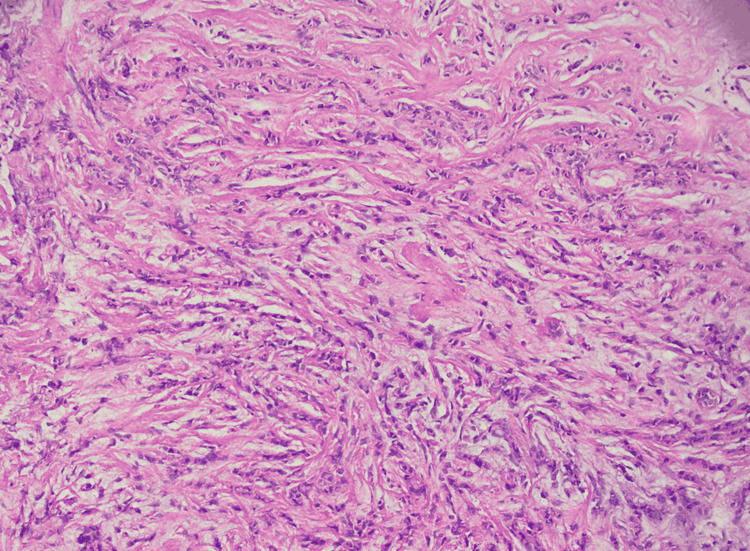
Histopathology invasive lobular carcinoma (HE x20)

**Figure 3:**
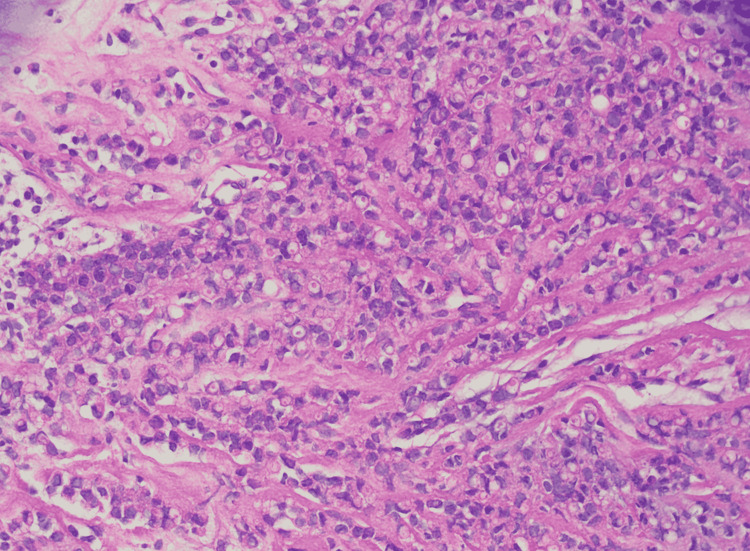
Histopathology invasive lobular carcinoma (HE x40)

The patient underwent an ultrasound-guided trucut biopsy of the left breast lump as the next step in the diagnostic algorithm. The histopathology report was consistent with grade two invasive lobular carcinoma as seen in Figures 2, 3. Ultrasound-guided fine-needle aspiration cytology from the left axillary lymph node showed atypical pleomorphic cells, suggestive of metastatic carcinoma. As it was clinically stage 3 invasive lobular carcinoma upon arrival to our outpatient department, she was advised to undergo Immuno-histochemical stains, CT chest/abdomen/pelvis with contrast, and MRI bilateral breast to define the extent of the disease.

**Figure 4:**
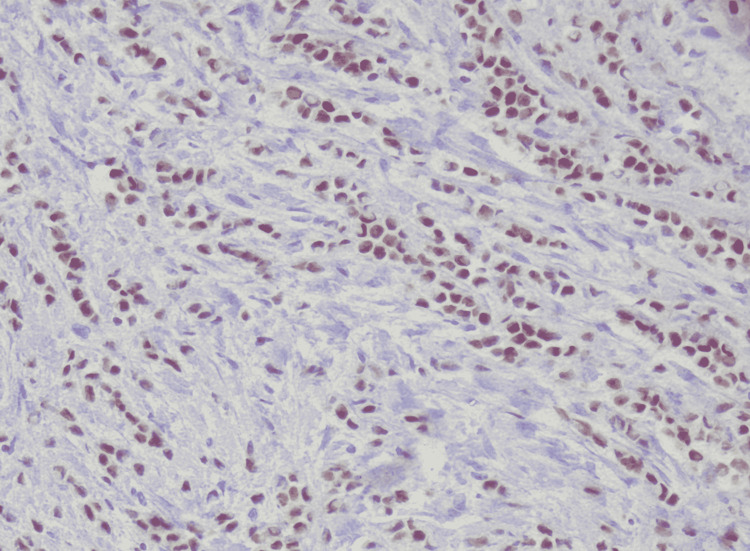
ER positive in 60% cells (HE x40)

**Figure 5:**
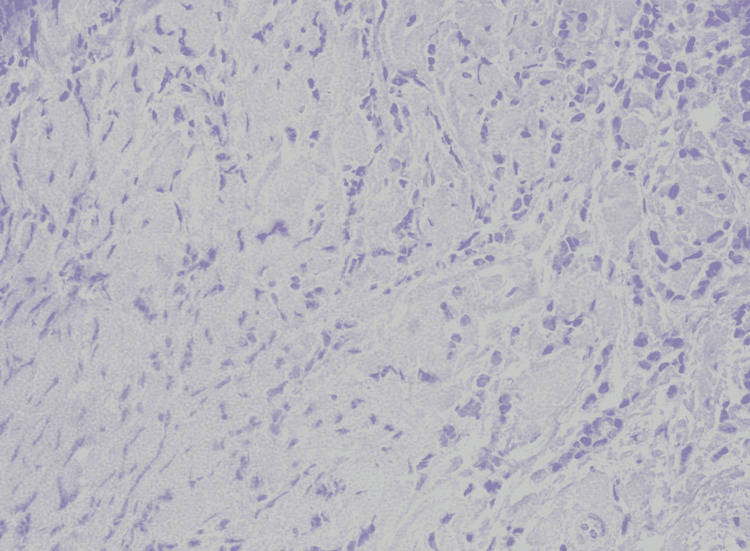
PR positive in 5% of cells (HE x40)

**Figure 6:**
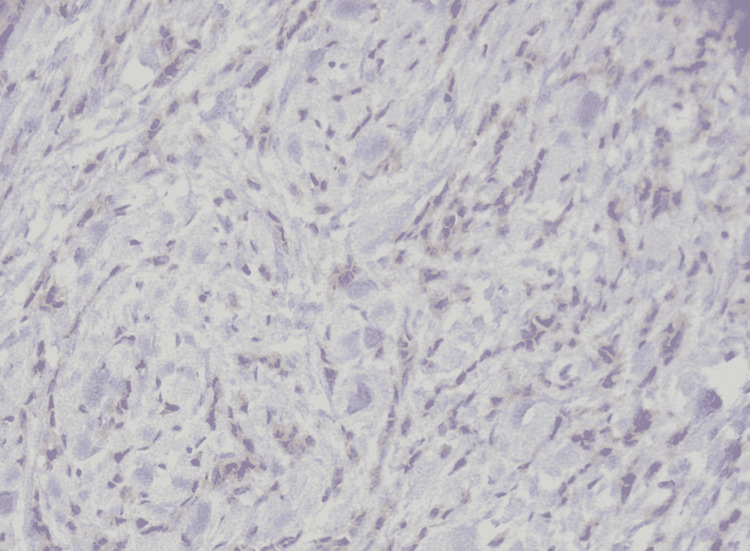
HER2/neu negative ( HE x40)

**Figure 7:**
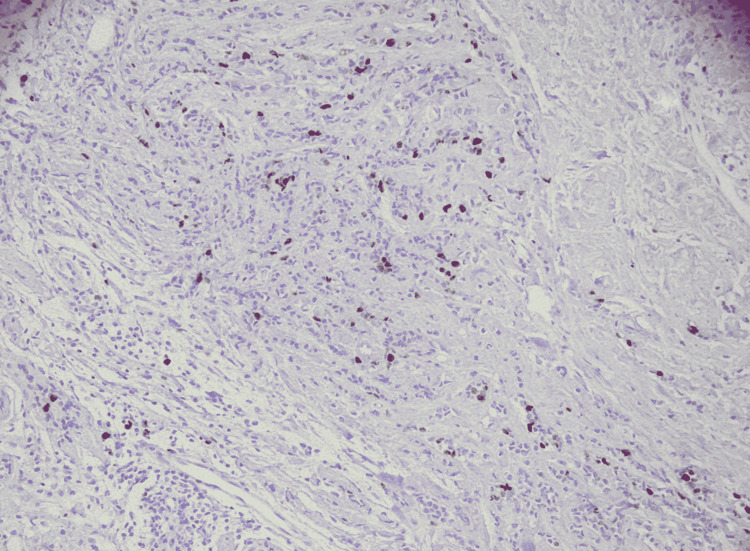
Ki-67 less than 10% (HE x40)

Immunohistochemical stains of neoplastic cells were performed where estrogen receptor (ER) was expressed in 60% of cells as seen in Figure 4, progesterone receptor (PR) was expressed in 5% of cell as seen in Figure 5, human epidermal growth factor receptor-2 (Her 2-neu) was present in greater than 10% of cells as seen in Figure 6 and Ki-67 labeling index was less than 10% as seen in Figure 7.

**Figure 8:**
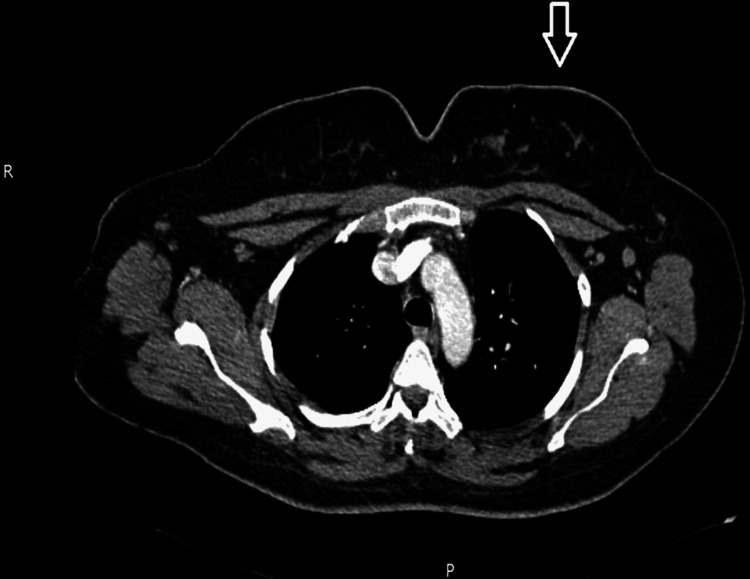
Computed tomography of chest, abdomen and pelvis with contrast was done, which showed the left breast having minimal parenchymal asymmetry

Subsequently, full staging with computed tomography of the chest, abdomen, and pelvis with contrast showed the left breast having minimal parenchymal asymmetry and a small 9mm node in the left axilla as seen in Figure 8. There was no evidence of metastases.

**Figure 9:**
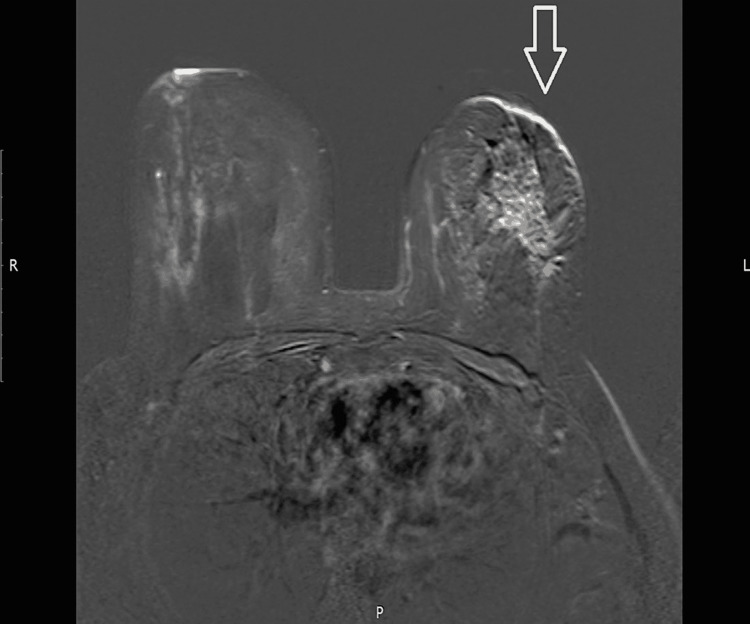
Magnetic resonance imaging shows left breast showed 4.2 x 3.3cm heterogeneously enhancing asymmetric mass like enhancement area within the left breast outer quadrant with adjacent another spiculated nodular lesion measuring 2.2cm

Her MRI left breast showed 4.2 x 3.3cm heterogeneously enhancing asymmetric mass-like enhancement area within the left breast outer quadrant with adjacent another spiculated nodular lesion measuring 2.2cm as seen in Figure 9, which was not evident on mammogram and ultrasound. The right breast showed few non-mass-like enhancement areas and small, non-enhancing subcentimeter bilateral axillary nodes. The overall impression was BIRADS I classification of right breast, which is considered normal, and BIRADS VI classification of left breast, which is proven neoplasm.

After a multidisciplinary approach, it was decided to give a trial of neoadjuvant chemotherapy to shrink the size of the tumor and prevent its further metastasis, followed by complete mastectomy of the left breast and radiation therapy. The decision to undergo mastectomy was based on the multi-centricity of the disease and the retro areolar nature of the tumor. She was started on Adriamycin and Cyclophosphamide as a part of neoadjuvant chemotherapy after explaining the side effects and duration of therapy. She will be followed up in regular intervals to monitor growth shrinkage and any untoward side effects.

## Discussion

Delay in diagnosis and treatment of cancer is an issue of concern among physicians for many years. According to Pack and Gallo delay on the part of the patient is considered reasonable when the time passed between the onset of symptoms and the first visit to a physician is under three months and it is considered an undue delay when this interval is three months or over. The subsequent delay includes turning down the physician's advice as well as a few cases without turning down the advice but with undue delay after consulting the physician, which makes the total delay from onset of symptoms to visit the clinic beyond the three months period [4]. In Pakistan, women approach health care facilities at the later stages of breast cancer due to multiple socio-cultural factors present in our society that prevent the woman from accessing health care at the onset of signs and symptoms [5]. In our case, the delay was one year after initial symptoms appeared and the socio-cultural factors that led to this were lack of awareness, religious misconceptions, stigmatization of breast cancer, reluctance to see a male physician, and the high cost of cancer treatment in Pakistan.

Lack of awareness

Lack of awareness and knowledge is associated with delayed presentation of breast cancer [6]. The delay in presentation results in an increase in tumor size, advanced stage, and worse prognosis. Patients with delayed presentation have lower survival rates when compared to those presenting early in the course of the disease [7]. In Pakistan, roughly half of the patients present at stage III or IV of breast cancer which is advanced and leads to a poor outcome [8].

Religious misconceptions

Religious misconceptions and myths are prevalent in Pakistan due to high illiteracy rates. People follow twisted religious versions and associate crisis and illness with God’s will and believe that only God can cure the disease [9]. As a consequence, people consult religious healers instead of visiting specialized physicians and this results in treatment delays [10].

Social stigma and taboo

Breast cancer is considered a social stigma and taboo in Pakistan. If a woman is diagnosed with breast cancer, friends and acquaintances keep a distance and view her as a bad and unlucky woman who is inflicted punishment by God for her bad deeds. The young woman hides their disease due to fear of social rejection and married women due to fear of divorce from their husband [9].

Reluctance to see a male physician

Pakistani women are raised in a culture where breasts are considered a body part that has to be covered all the time. Such behavior and practices are considered modest and virtuous behavior in a woman. Due to such upbringing majority of women do not want their breasts to be examined by male physicians. They feel unprotected from being examined by male physicians and many women even get embarrassed if a breast examination is done by a female physician [11].

High cost of cancer treatment

In Pakistan, there are fewer cancer screening and treatment facilities and patients usually avoid treatment due to poverty. The social structure of Pakistan provides fewer opportunities for women to be financially independent and make them rely on their husband’s wealth which leads to financial insecurity and delays in expensive breast treatment [9]. There are few studies from Pakistan that state that poor economic and financial conditions are significant barriers to breast cancer screening and treatment [12,13].

The consequences of delay as in our case were to pay for the high cost of cancer treatment due to stage 3 and to undergo the surgical procedure of mastectomy. They both have a significant impact on the patient’s psychology and finances. It is estimated that the later the stage the higher the cost of breast cancer treatment and in stage 4 of a breast cancer patient is expected to pay approximately 50% more for the breast cancer treatment compared to earlier stages [14]. Women with mastectomy have reported significantly reduced femininity and emotional strain due to missing body parts [15]. Loss of morale caused by mastectomy may lead to tensions in their sexual life, marital life, and interpersonal relationships. Patients also find it difficult to get back to their social life [16]. Body image disfigurements and sexual disturbances caused by mastectomy can lead to increased rates of depression [17]. The psychological impacts are greater in younger women than in older women [18]. Younger women also want breast reconstruction after mastectomy more than older women which indicates that the impacts of mastectomy are more pronounced in younger women [19].

## Conclusions

This case highlights some of the important socio-cultural factors prevalent in Pakistan that lead to delays in the diagnosis and treatment of breast cancer. This results in an overall poor prognosis as discussed above. In our case, the outcome would have been better if the patient sought medical attention at the onset of the signs and symptoms of the disease. Therefore, we propose educational interventions via newspaper, social media, educational syllabus, and television to educate the masses and raise awareness among them about the importance of early intervention. The aim should be to reach the community level and door-to-door spread of message with the help of lady health workers. We should also involve religious scholars in educating people as the majority of people in Pakistan consider highly their advice and thought processes about the disease. Our healthcare policymakers should make strategies to bring down the financial cost of breast cancer treatment and encourage women to take initiative in health care. With these interventions, we can somehow control the delayed diagnosis and grave consequences of breast cancer among women in Pakistan.

## References

[1] McCart Reed AE, Kutasovic JR, Lakhani SR, Simpson PT (2015). Invasive lobular carcinoma of the breast: morphology, biomarkers and 'omics. Breast Cancer Res.

[2] (2022). Breast Cancer: Statistics. https://www.cancer.net/cancer-types/breast-cancer/statistics.

[3] Sohail S, Alam SN (2007). Breast cancer in Pakistan-awareness and early detection. J Coll Physicians Surg Pak.

[4] Pack GT, Gallo JS (1938). The culpability for delay in treatment of cancer. Am J Cancer.

[5] Hameed Khaliq I, Mahmood HZ, Sarfraz MD, Masood Gondal K, Zaman S (2019). Pathways to care for patients in Pakistan experiencing signs or symptoms of breast cancer. Breast.

[6] Adebamowo CA, Ajayi OO (2000). Breast cancer in Nigeria. West Afr J Med.

[7] Richards MA, Smith P, Ramirez AJ, Fentiman IS, Rubens RD (1999). The influence on survival of delay in the presentation and treatment of symptomatic breast cancer. Br J Cancer.

[8] Khokher S, Qureshi MU, Mahmood S, Sadiq S (2016). Determinants of advanced stage at initial diagnosis of breast cancer in Pakistan: adverse tumor biology vs delay in diagnosis. Asian Pac J Cancer Prev.

[9] Saeed S, Asim M, Sohail MM (2021). Fears and barriers: problems in breast cancer diagnosis and treatment in Pakistan. BMC Womens Health.

[10] Shamsi U, Khan S, Azam I (2020). Patient delay in breast cancer diagnosis in two hospitals in Karachi, Pakistan: preventive and life-saving measures needed. JCO Glob Oncol.

[11] Naz N, Khanum S, Dal Sasso GT, de Souza ML (2016). Women’s views on handling and managing their breast cancer in Pakistan: a qualitative study. Diseases.

[12] Gulzar F, Akhtar MS, Sadiq R, Bashir S, Jamil S, Baig SM (2019). Identifying the reasons for delayed presentation of Pakistani breast cancer patients at a tertiary care hospital. Cancer Manag Res.

[13] Khan MA, Hanif S, Iqbal S, Shahzad MF, Shafique S, Khan MT (2015). Presentation delay in breast cancer patients and its association with sociodemographic factors in North Pakistan. Chin J Cancer Res.

[14] Blumen H, Fitch K, Polkus V (2016). Comparison of treatment costs for breast cancer, by tumor stage and type of service. Am Health Drug Benefits.

[15] Shrestha K (2012). Psychological impact after mastectomy among Nepalese women: a qualitative study. Nepal Med Coll J.

[16] Schmauss AK, Kirsch A, Schönhof P (1985). Psychological and social behavior of females following radical operation of breast cancer (Article in German). Zentralbl Chir.

[17] Reich M, Lesur A, Perdrizet-Chevallier C (2008). Depression, quality of life and breast cancer: a review of the literature. Breast Cancer Res Treat.

[18] Lee CN, Foster RD (2005). Breast reconstruction after mastectomy in young women. Breast Dis.

[19] Fang SY, Balneaves LG, Shu BC (2010). "A struggle between vanity and life": the experience of receiving breast reconstruction in women of Taiwan. Cancer Nurs.

